# Supplementary material for: Selenium May Be Involved in Esophageal Squamous Cancer Prevention by Affecting GPx3 and FABP1 Expression: A Case-Control Study Based on Bioinformatic Analysis
Source: Nutrients. 2024 Apr 28;16(9):1322. doi: 10.3390/nu16091322 (PMC11085500; doi:10.3390/nu16091322)
Supplement: Supplementary file 1 [file nutrients-16-01322-s001.zip › nutrients-2944672-supplementary.pdf]

Article

Selenium May Be Involved in Esophageal Squamous Cancer Prevention by Affecting GPx3 and FABP1 Expression: A Case-Control Study Based on Bioinformatic Analysis

Niannian Wang [1], Da Pan [1], Xiaopan Zhu [1], Xingyuan Ren [1], Xingyi Jin [1], Xiangjun Chen [1,2], Yuanyuan Wang [1], Ming Su [3], Guiju Sun [1] and Shaokang Wang [1,2,*]

[1] Key Laboratory of Environmental Medicine and Engineering, Ministry of Education, Department of Nutrition and Food Hygiene, School of Public Health, Southeast University, Nanjing 210009, China; wangnian-nian@foxmail.com (N.W.); pantianqi92@foxmail.com (D.P.); zhuxiaopandhr@163.com (X.Z.); mn13451537048@126.com (X.R.); xingyijin@foxmail.com (X.J.); cxjll910@163.com (X.C.); 230218460@seu.edu.cn (Y.W.); gjsun@seu.edu.cn (G.S.)

[2] Department of Public Health, School of Medicine, Xizang Minzu University, Xianyang 712000, China

[3] Huai'an District Center for Disease Control and Prevention, Huai'an 223001, China; suminglala@163.com

* Correspondence: shaokangwang@seu.edu.cn; Tel.: +86-153-1206-0480

Table S1. Differential genes screened from tissues with low expression of GPx3

	DEGs
Downregulated genes (N=121)	CRNN, MUC21, KRT4, BPIFB2, TGM3, CRISP3PGA5, DYNAP, LIPF, MAL, KRT78, TMPRSS11B, IL36A, SPINK7, KRT13, CAPN14, ATP4B, FABP4, PGA3, CLCA4, GYS2, SLURP1, DEFB4B, CLDN17, SPINK5, MUC22, SPRR3, NR0B1, GKN1, ARSF, LCE3C, KRT24, ATP4A, PRSS27, RHCG, SPRR2F, LCE2B, CRCT1, SCEL, SCGB3A1, ATP12A, PI16, ENDOU, CWH43, VSIG8, ACER1, HS3ST4, FOXI2, SST, TFAP2B, SERTM1, SERPINB12, LCE2A, KLK13, FMO2, OTX2, CIDEA, PAX7, LCE6A, NCCRP1, SCNN1B, S100A7, SPINK8, TGM6, SCARA5, TMPRSS11A, TMPRSS11E, CBLIF, FLG2, CRTAC1, FGFBP2, KRT1, SPRR2A, UPK1A, SPRR2B, PNLDC1, TMEM155, TGM5, IGFL1, S100A12, LORICRIN, PADI1, ADH1B, AADACL2, MAB21L3, PAX1, LEXM, KRT77, TCHH, IRX1, OTOP2, KRT79, A2ML1, SERPINB3, PPP1R3C, SPRR2G, NHLH2, CCN5, HMX1, LCE3E, REG1B, TBC1D3, RNASE7, CNFN, LYNX1, CYSRT1, SPRR2D, CMA1, DEFA6, NLRP10, SERPINB4, FLG, CENPVL3, NXPE4, ATP6V0A4, KPRP, GBP6, PPL, KRT2, FOXI3, PNPLA5
Upregulated genes (N=44)	MSLNL, SPINK4, KCNQ2, TRIM54, HBE1, HSD3B2, FOXH1, TAAR6, MTTP, GATA4, SPANXC, SEMG2, SLC2A2, CALHM3, CDH18, ITLN1, MUC12, OR51B4, **FABP1**, CPS1, AC009163.3, PAX4, OTC, CDH9, ALB, SEMG1, DAZ2, DMBT1, FAM133A, TPTE, FSTL5, TMEM207, TSPY2, NTSR1, CLCA1, CRYGD, MAGEB1, AQP2, PPP1R1B, PAGE2, GC, LEFTY1, CCL25, DCAF4L2

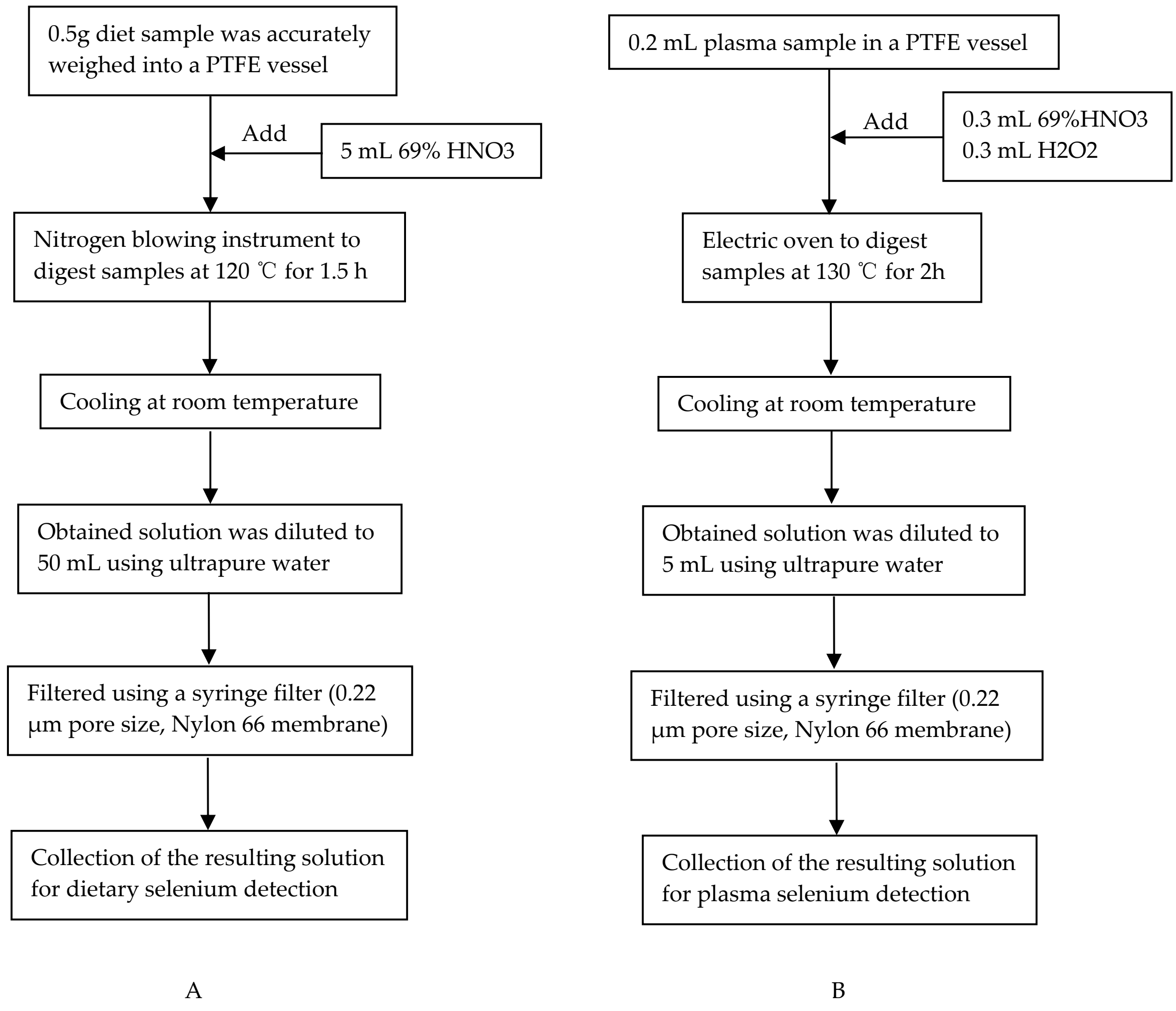

Figure S1 Flowchart of sample pretreatment for detection of A, dietary selenium; B, plasma selenium level.
